# A Novel UHPLC-MS Method Targeting Urinary Metabolomic Markers for Autism Spectrum Disorder

**DOI:** 10.3390/metabo10110443

**Published:** 2020-11-02

**Authors:** Dominika Olesova, Jaroslav Galba, Juraj Piestansky, Hana Celusakova, Gabriela Repiska, Katarina Babinska, Daniela Ostatnikova, Stanislav Katina, Andrej Kovac

**Affiliations:** 1 Institute of Neuroimmunology, Slovak Academy of Sciences, Dubravska Cesta 9, 84510 Bratislava, Slovakia; dominika.olesova@savba.sk; 2 Department of Pharmaceutical Analysis and Nuclear Pharmacy, Faculty of Pharmacy, Comenius University in Bratislava, Odbojarov 10, 832 32 Bratislava, Slovakia; jaroslav.galba@gmail.com (J.G.); piestansky@fpharm.uniba.sk (J.P.); 3 Institute of Physiology, Faculty of Medicine, Comenius University in Bratislava, Sasinkova 2, 813 72 Bratislava, Slovakia; hana.celusak@gmail.com (H.C.); gabika.repiska@gmail.com (G.R.); katarina.babinska@fmed.uniba.sk (K.B.); daniela.ostatnikova@fmed.uniba.sk (D.O.); 4 Institute of Mathematics and Statistics, Faculty of Science, Masaryk University, Kotlářská 267/2, 611 37 Brno, Czech Republic; katina@math.muni.cz

**Keywords:** autism spectrum disorder, liquid chromatography, mass spectrometry, metabolomics, oxidative stress, gut microbiota

## Abstract

Autism spectrum disorder is a heterogeneous neurodevelopmental disease. Currently, no biomarker of this disease is known. Diagnosis is performed through observation, standardized behavioral scales, and interviews with parents. In practice, diagnosis is often delayed to the average age of four years or even more which adversely affects a child’s perspective. A laboratory method allowing to detect the disorder at earlier stages is of a great need, as this could help the patients to start with treatment at a younger age, even prior to the clinical diagnosis. Recent evidence indicates that metabolomic markers should be considered as diagnostic markers, also serving for further differentiation and characterization of different subgroups of the autism spectrum. In this study, we developed an ultra-high performance liquid chromatography-tandem triple quadrupole mass spectrometry method for the simultaneous determination of six metabolites in human urine. These metabolites, namely methylguanidine, *N*-acetyl arginine, inosine, indole-3-acetic acid, indoxyl sulfate and xanthurenic acid were selected as potential biomarkers according to prior metabolomic studies. The analysis was carried out by means of reversed-phase liquid chromatography with gradient elution. Separation of the metabolites was performed on a Phenomenex Luna^®^ Omega Polar C18 (100 × 1.0 mm, 1.6 µm) column at a flow rate of 0.15 mL/min with acetonitrile/water 0.1% formic acid aqueous as the mobile phase. The analysis was performed on a group of children with autism spectrum disorder and age-matched controls. In school children, we have detected disturbances in the levels of oxidative stress markers connected to arginine and purine metabolism, namely methylguanidine and *N*-acetylargine. Also, products of gut bacteria metabolism, namely indoxyl sulfate and indole-3-acetic acid, were found to be elevated in the patients’ group. We can conclude that this newly developed method is fast, sensitive, reliable, and well suited for the quantification of proposed markers.

## 1. Introduction

Autism spectrum disorder (ASD) is a neurodevelopmental condition characterised by core symptoms that include impaired social interaction and communication skills, as well as repetitive patterns of behaviour and interests. ASD is connected with **a** heterogeneous presentation of symptoms with a high variability of cognitive functions ranging from severe intellectual disability to superior intelligence. Current rates of ASD prevalence in the population are estimated at 1.69% with male predominance [1,2,3]. The clinical heterogeneity of autism has created a need for better diagnostic approaches as the classification of the spectrum is still and the ongoing pursuit.

At the present time, no biomarker of ASD is known and the diagnosis of ASD is based on standardized behavioral scales obtained through the observation of patients performing a set of activities and social tasks, accompanied by parental interviews [2]. It has been shown that patients who received early medical intervention had better language and educational outcomes in the future. On the other hand, diagnosis at an advanced age and thus late access to therapies and interventions has adverse consequences on the long-term developmental outcomes, and it puts children at risk of requiring more special education and having worse socialization. For this reason, decreasing the average age of ASD diagnosis is currently a great challenge [4]. Laboratory biomarkers that could be quantitatively measured might be a chance to identify the risk, to provide earlier and more reliable diagnoses along with further differentiation of the autism spectrum according to common pathophysiological features allowing for individualised treatment and response monitoring.

A significant number of the recent metabolomic studies concerning ASD were performed on urine samples, which may be due to complications in blood sampling from children, even more difficult in those with severe forms of ASD [5]. Thanks to the noninvasive collection and relatively large volumes available, urine is a fundamental biological matrix in the metabolomic analysis. However, large variations in pH, osmolarity, and ionic strength together with vast concentration ranges complicate the analysis. Still, urine metabolome being a waste product perfectly reflects the current condition of the organism [6].

The aim of our recent study was to develop and validate an ultra-high performance liquid chromatography-tandem triple quadrupole (UHPLC-QqQ) mass spectrometry method for the simultaneous determination of methyl guanidine, *N*-acetyl arginine, inosine, indole-3-acetic acid, indoxyl sulfate, and xanthurenic acid that were selected as potential biomarkers for ASD according to prior metabolomic studies. The developed method was subsequently used to validate new potential urinary biomarkers on a sample of Slovak children with ASD and age-matched controls.

## 2. Results and discussion

### 2.1. Method Development

All analytes were detected under MS/MS conditions in MRM mode. Precursor ions and characteristic precursor ion to product ion transitions for both analytes and internal standards were identified from mass spectra acquired by direct infusion of standard solutions into the mass spectrometer. The optimal ion intensity of precursor and product ions was manually optimized by adjusting cone voltage (CV), and collision energy (CE) needed for ion fragmentation. These parameters were tested in the range from 5 to 50 V which was empirically found to be satisfactory for all analytes. Small analytes such as these tend to undergo intrasource fragmentation under higher voltages. The product ions for MRM transitions were chosen according to their intensity. Multi-reaction monitoring transitions used in the quantification method are listed in Table 1.

To achieve good separation necessary for selective and sensitive quantification several different columns were investigated under the reverse phase and HILIC mode. HILIC columns were tested with an aqueous mobile phase consisting of 10 mM ammonium formate + 0.1% formic acid and acetonitrile as an organic mobile phase. Two columns were tested, namely Acquity BEH Amide (100 × 2.1 mm, 1.7 µm) and YMC-Triart Diol (100 × 2.0 mm, 1.9 µm) columns. However, none provided satisfactory peak shapes for all analytes. Acquity CSH C18 (100 × 2.1 mm, 1.6 µm), Cortecs T3 (100 × 2.1 mm, 1.6 µm), BEH Shield RP18 (100 × 2.1 mm, 1.7 µm), and Phenomenex Luna^®^ Omega Polar C18 (100 × 1.0 mm, 1.6 µm) were among the columns tested under reverse phase mode. The mobile phase consisted of 0.1% formic acid in the aqueous phase and acetonitrile in the organic phase. The best results were achieved with reverse phase column Phenomenex Luna^®^ Omega 1.6 µm Polar C18 (100 × 1.0 mm) which was later used in the analysis (Supplementary Figure S1). Although, the separation of analytes was better using Shield RP18 compared to Polar C18 column, when we compared the signal to noise ratio Polar C18 provided better results (Supplementary Figure S2). Also, a smaller diameter of Polar C18 provides increased signal intensity for all analytes and thus is more suitable for the analysis of selected compounds.

**Figure 1:**
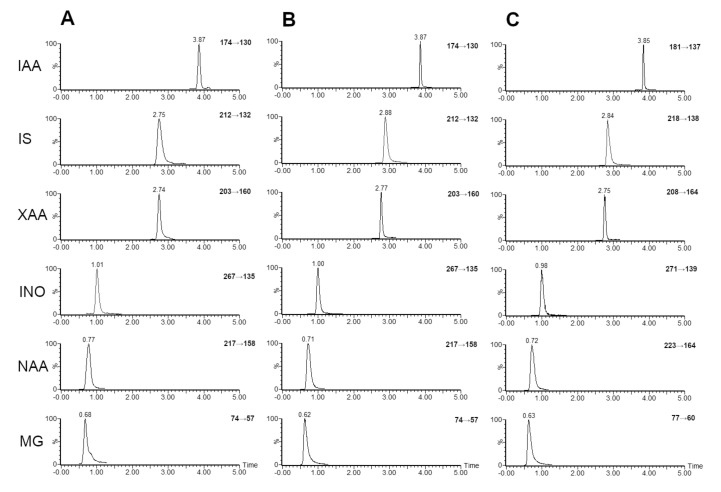
Representative chromatograms of methylguanidine (MG), *N*-acetylarginine (NA), inosine (INO), xanthurenic acid (XAA), indoxyl-sulphate (IS) and indole-3-acetic acid (IAA) of urine sample (**A**), standard solution (**B**) and internal standards (**C**).

Gradient elution mode was selected for optimal separation of analytes with regard to minimizing the run time. The retention times for analytes targeted in this study were 0.63 min for methylguanidine, 0.75 min for *N*-acetylarginine, 1.00 min for inosine, 2.73 min for xanthurenic acid, 2.79 min for indoxyl sulfate and 3.85 min for indole-3-acetic acid as displayed on Figure 1. With the total run time being 8.5 min, this method provides a quick analysis of large sets of samples. To achieve good intensity and separation 0.1% formic acid in water was used as aqueous mobile phase A and pure acetonitrile as organic mobile phase B.

Different approaches were tested concerning sample preparation. Freeze-drying or lyophilization was tested as a possible method for the pre-concentration of samples. However, the results were not satisfactory due to nonlinear behaviour of indoxyl sulfate and *N*-acetylarginine signal and thus was considered not suitable for the analysis of all compounds (Supplementary Figure S3). One of the most often used extraction techniques in LC-MS applications—solid phase extraction (SPE) was also subjected to the testing. Due to the contrasting chemical properties of the studied compounds the selection of sorbent was important. For this purpose, we have tested OASIS PRIME HLB 30 mg and Oasis MCX 30mg (Waters, Prague, Czech Republic). Oasis Prime HLB was selected for its enhanced retention for polar compounds and Oasis MCX (the mixed mode—reverse phase + cation exchange) was selected for the possibility of high retention of acids, bases and neutrals at once in controlled pH load and elution. However, when compared to 1:2 dilution in the mobile phase, SPE sorbents did not provide better recoveries (Supplementary Table S1). Simple dilution was assessed in ratios of 1:1 and 1:2 with the best results achieved at a dilution of 1:2 (Supplementary Table S2) Therefore, the dilution in ratio 1:2 was selected as a suitable method for sample preparation.

### 2.2. Method Validation

The linearity of the developed method was tested in ranges from 12 to 2400 ng/mL for inosine and xanthurenic acid, from 20 to 4000 ng/mL for indole-3-acetic acid and from 40 to 8000 ng/mL for methylguanidine, *N*-acetylarginine and 480 to 48 000 ng/mL for indoxyl sulfate. Although urine concentration is substantially unpredictable and influenced by fluid intake, these ranges are sufficient to cover physiological and pathological concentration levels in children’s urine. The linear regression equations, experimental linear ranges, coefficients of determination (r^2^), limits of detection (LODs), and limits of quantification (LOQs) are summarized in Table 2.

Deuterated internal standards of each analyte were used for the construction of calibration curves, QC samples and patients’ samples to minimize the influence of systematic bias and matrix effects. Precision and accuracy were evaluated by analysis of QC samples prepared in the sample matrix across three concentration levels. The intra-day precision (RSD%) for all analytes ranged from 0.26 to 10.10%, and the corresponding accuracy (% relative error) was within 92% to 106% for all analytes except methylguanidine. For this analyte, accuracy ranged from 61% to 72% with relative standard deviation ranging from 0.28% to 1.15%. This was probably caused by matrix effects affecting the internal standard less than analyte as linearity for this compound was considerably high with R2 = 0.99958. Concerning inter-day assay, precision ranged between 2.17% to 14.84% and accuracy ranged from 91% to 105% for all analytes except methylguanidine which inter-day accuracy was between 61% to 76% with precision from 12.39% to 13.30%. The results are summarized in Supplementary Table S3 and present satisfactory quality assurance for method accuracy and precision. The matrix effects were evaluated by comparing peak areas of internal standards in urine samples and standard solutions. These ranged between 11% to 83%, which indicates a strong influence of the matrix on the ionization of analytes (Supplementary Table S4). This issue is accounted for by the use of deuterated internal standards, which compensate for the matrix effects. The stability of the analytes stored in autosampler over 24 h and freeze/thaw stability are summarized in Supplementary Table S5. All of the analytes were stable in autosampler for 24h with values within the acceptance limits of accuracy (±15% RE) and precision (±15% CV). The results of stability studies indicate that all standards except of xanthurenic acid and indoxyl sulfate were stable during repeated freeze/thaw conditions. Thus, the samples for analysis should always be prepared from freshly thawed samples and analyzed immediately after sample preparation.

### 2.3. Analysis of Urine Samples

**Figure 2:**
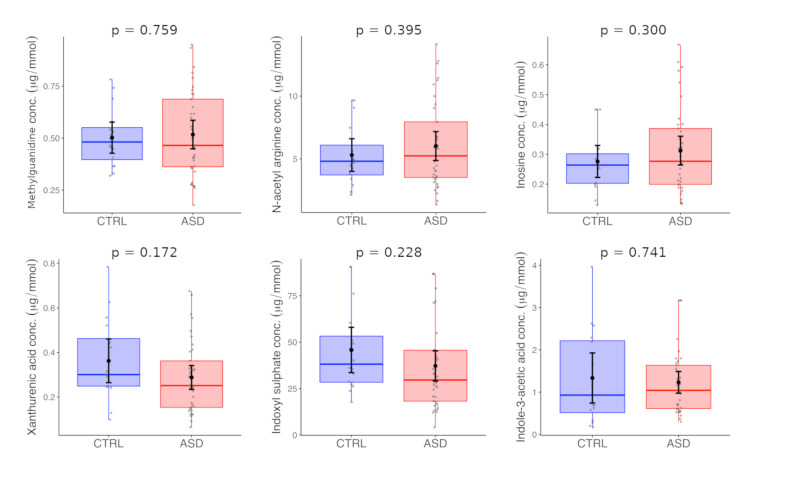
Analysis of urine samples of preschool-children with ASD. The optimized UHPLC-MS/MS method was applied for the determination of MG, NAA, INO, XAA, IS, and IAA in the urines of ASD children and age-matched control group.

**Figure 3:**
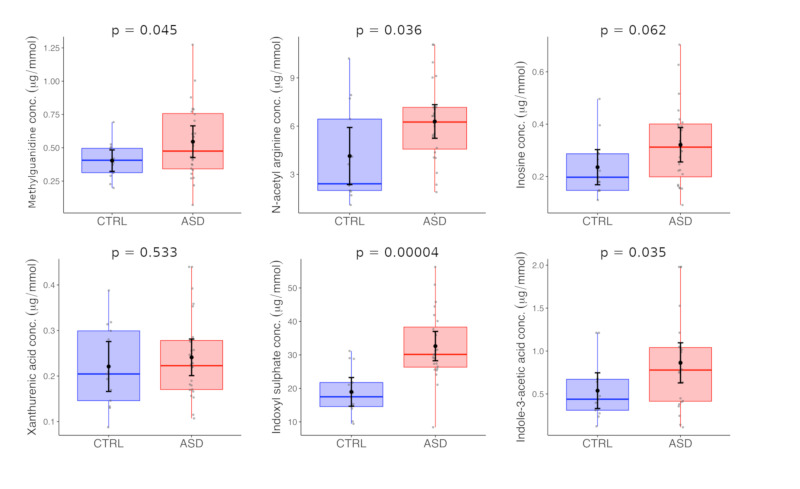
Analysis of urine samples of school children with ASD. The optimized UHPLC-MS/MS method was applied for the determination of MG, NAA, INO, XAA, IS, and IAA in the urines of ASD children and age-matched control group.

The described method was used for the analysis of urine samples of Slovak children with ASD and typically developing age-matched controls. Firstly, we compared the mean levels of studied analytes between the two populations (ASD vs. controls). No significant differences were found. According to our data, we assume that it could be caused by age heterogeneity of the tested set of participants involved in our study. The groups were subsequently divided according to age into two subgroups: the pre-school children (<6 years) (Figure 2) and school-children (≥6 years) (Figure 3).

The statistical evaluation of these data is listed in Table 3. Furthermore, we have found that neither dietary restrictions nor gastro-intestinal disturbances have any impact on metabolite levels (Table S6).

We have detected elevated levels of indoxyl sulfate (mean 18.95 ± 7.11 µmol/mmol Cr in CTRL vs. 32.63 ± 10.38 µmol/mmol Cr in ASD, *p* = 0.00004), methylguanidine (mean 0.4 ± 0.13 µmol/mmol Cr in CTRL vs. 0.55 ± 0.28 µmol/mmol Cr in ASD, *p* = 0.045), *N*-acetyl arginine (mean 4.14 ± 2.94 µmol/mmol Cr in CTRL vs. 6.29 ± 2.48 µmol/mmol Cr in ASD, *p* = 0.036) and indole-3-acetic acid (mean 0.54 ± 0.34 µmol/mmol Cr in CTRL vs. 0.86 ± 0.55 µmol/mmol Cr in ASD, *p* = 0.035) in patients with ASD compared to the typically developing children. All these changes were found only in school children and we did not detect them in pre-school group.

A variety of biochemical markers have been proposed for ASD and thorough validations need to be performed to assess the correlation between different pathological features and proposed biomarkers [7]. Metabolomics is currently being an important issue in biomarker discovery. Small molecule and lipid screening is a promising strategy for the discovery of markers for diagnostics and disease progress tracking but also elucidating the biochemical processes and phenotype investigations [8]. There is evidence that some metabolic disorders are more frequent in ASD patients. Among these disorders are phenylketonuria, creatine deficiency syndromes, adenylosuccinate lyase deficiency or purine/pyrimidine metabolism disorders. Interestingly, previous studies showed that mothers of children with ASD share some common metabolic disturbances with their children. The origin of these abnormalities remains still unclear but it points the interest into the elucidation of biochemical processes that seem to be imbalanced in ASD patients [9,10]. Typically, metabolome explorations start with untargeted profiling of biofluids and tissues. Concerning urinary metabolome of ASD patients, a number of such studies have been performed with identifications of several implicated metabolic pathways. Although few urinary metabolites have changed consistently among these studies, there is consensus in the implicated biochemical pathways [5]. The most significant changes were found in pathways connected with gut microbiota, tryptophan, purine, aminoacid metabolism, neurotransmitters and elevated markers of oxidative stress [9,11,12,13,14,15,16].

Methylguanidine together with inosine and *N*-acetylarginine are assumed to accumulate as a result of excessive oxidative processes in living organisms reflecting changes in arginine/creatine metabolism and purine metabolic pathway [17,18]. The role of oxidative stress in the pathogenesis of ASD was repeatedly described by many researchers [19,20,21]. Increased markers of oxidative stress are linked to the dysfunction of energy metabolism in mitochondria, which may clinically manifest with features similar to ASD. Among the mostly observed are glutathione metabolism, purine metabolites, lipid peroxidation products and nitric oxide/arginine metabolism [22,23]. In our study, we have found slightly elevated levels of *N*-acetylargnine in school children. Also, our data revealed a perturbation in the levels of methylguanidine, a known uremic toxin. However, this is in contrast with findings of Diémé et al., where decreased levels were detected [9]. With central nervous system being particularly sensitive to reactive oxygen species this indicates that oxidative stress may be implicated not only during the early stages of development but also contributes to pathophysiology [24].

Currently, the gut-brain axis is widely discussed concerning ASD as many patients have reported GIT problems. According to a meta-analysis, gastrointestinal dysfunction is more than three times more prevalent in ASD [25] and correlate with the severity of neurological symptoms [26,27]. Differences in the composition of the gut microbiota have been observed in ASD, and the altered populations of bacteria produce specific metabolites than can be absorbed into the circulation and introduce changes into normal biochemical processes [28]. Some of the compounds that were found to be altered in ASD, are known uremic toxins (e.g., methylguanidine, indoxyl sulfate, indole-3-acetic acid, p-cresol, hypoxanthine), and their elevated levels may cause neuronal damage [29]. A group of indole derivatives—products of tryptophan metabolism by gut bacteria but not mammalian cells—are included in this group. In this study, we have found elevated levels of indoxyl sulfate and indole-3-acetic acid in ASD group, which is in agreement with previous findings [9,14]. It is important to note that we were able to identify these changes solely in the group of school children. This opens the question whether pathological processes connected with these metabolites are progressing with age from birth or appear only later in life.

Among the potential limitations of our study is the small sample size, results should be replicated in larger cohorts. The male prevalence of ASD implicates that the sex of patients may contribute to the phenotype and should be considered for the next studies as well. Patients with ASD may often have different eating habits and therefore dietary behavior should be included in the next studies.

## 3. Materials and Methods

### 3.1. Study Participants

The sample involved 90 children (all boys), of that 61 individuals with ASD aged 4.5 ± 1.8 years (mean ± standard deviation, SD) and 29 control boys aged 5.7 ± 2.1 years (mean ± SD). The diagnostic process comprised two diagnostic methods Autism Diagnostic Observation Schedule 2nd revision (ADOS-2) [30] and Autism Diagnostic Interview-Revised (ADI-R) [31] which are considered to be “golden standard” diagnostic tools for ASD assessment. Diagnostic procedures were performed by trained clinical psychologists and the decision was made after consensus in clinical judgment. Based on age, the sample was divided into two groups. The younger age group included 37 preschool children with ASD aged less than 6 years, average age 4.1 ± 0.8 (mean ± SD), the total calibrated score ADOS-2 was 7.6 ± 1.8 (mean ± SD). The age-matched control group involved 16 children aged 4.7 ± 0.7 (mean ± SD). The older age group included 24 school children with ASD (all boys) aged 6.0–10.0 years (7.7 ± 0.9, mean ± SD). The total calibrated score ADOS-2 was 7.7 ± 0.9 (mean ± SD), the age-matched control group involved 13 children aged 8.2 ± 1.2 (mean ± SD). All experiments with human samples were monitored by the Ethical committee of the Comenius University Faculty of Medicine, and the University hospital in Bratislava, Slovakia and it is consistent with the 1964 Helsinki declaration and its later amendments. Parents were aware of the whole design of the study and informed consent form was signed by both (or at least one if both are not available) parents or caregivers of the corresponding child.

### 3.2. Chemicals and Materials

Methylguanidine, *N*-acetyl arginine, inosine, indole-3-acetic acid, indoxyl sulfate, xanthurenic acid, formic acid, LC/MS grade acetonitrile and methanol were purchased from Sigma-Aldrich (St. Louis, MO, USA). Internal standards D7-indole-3-acetic acid, N4-inosine, and C6-indoxyl sulfate were purchased from Cambridge Isotope Laboratories Inc., D3-methylguanidine and D4-xanthurenic acid from Santa Cruz Biotechnology Inc., and D7-*N*-acetyl arginine from ALSACHIM (Illkirch Graffenstaden, FR). All standards were of analytical grade. Water was purified using a Millipore purification system (Bedford, MA, USA).

### 3.3. Preparation of Standards

Stock solutions of indoxyl sulfate, methylguanidine and inosine were prepared by dilution in water, *N*-acetyl arginine was prepared with the addition of 100 µL of 1 mol/L HCl, and stock solutions of indole-3-acetic acid and xanthurenic acid were prepared with the addition of 100 µL of 2 mol/L NaOH. All stock solutions were prepared at concentration 1mg/mL and kept at −20 °C until use. The working solutions were freshly prepared from the frozen stock solution for every analysis. Calibration curves and quality control samples were prepared by diluting the stock solutions with 0.1% formic acid in water-acetonitrile (95:5).

### 3.4. Mass Spectrometric Conditions

LC-MS/MS analysis was performed on ACQUITY UPLC I-Class coupled with triple quadrupole mass spectrometer ACQUITY XEVO TQD (Waters, Prague, Czech Republic) with electrospray ionization (ESI) source in both positive and negative mode. MS/MS analysis was performed in the multiple reaction monitoring (MRM) mode. The electrospray capillary voltage was set to 2.0 kV, desolvation gas flow was 650 l/hr with a temperature 350 °C, and the source temperature was set to 150 °C. MassLynx software version 4.1 (Waters) was used for the data acquisition, and TargetLynx XS (Waters) was used for processing.

### 3.5. Chromatographic Conditions

Chromatographic separation was carried out on Phenomenex Luna Omega Polar C18 (100 × 1.0 mm, 1.6 µm particles) in a reverse phase mode with gradient elution. Mobile phase A consisted of 0.1% formic acid in the water, and mobile phase B consisted of 100% acetonitrile. The elution started at 5% B (0–1.0 min.) increasing to 55% B (1.0–3.5 min.) then to 95% B (3.5–5.0 min), returning to 5% and eventually re-equilibrating from 5.0 to 7.5 min. The column temperature was set to 40 °C with a flow rate of 0.15 mL/min and the injection volume 5 µL.

### 3.6. Sample Preparation

Standard solutions for mass spectrometric tuning were prepared from freshly thawed stock solutions of each analyte diluted to 10 µg/mL in 50% methanol + 0.1% formic acid. Calibration standards were prepared from freshly thawed stock solutions by dilution in starting mobile phase composition—water + 0.1% formic acid/acetonitrile (95:5). Urine samples for quantification were prepared as follows. First, urine samples were centrifuged at 30,000× *g* for 10 min. Then, 50 µL of supernatant was spiked with the solution of internal standards and diluted to volume 150 µL with water. Samples were then transferred to vials and subjected to LC-MS analysis.

### 3.7. Method Validation

The developed analytical method was validated for linearity, sensitivity, precision, accuracy and stability. Linearity was evaluated by performing calibration curves using seven standards. The calibration equation was generated as a linear function (y = ax + b) in Targetlynx XS software using the stable isotope-labeled internal standards. Analyte concentrations were calculated as the ratio of analyte peak area over the internal standard peak area and compared with the calibration curve. Limits of quantitation and detection were quantitated from the calibration equation as 3 × SD a/b for LOD and 10 × SD a/b for LOQ. Precision and accuracy were determined by the quantitation of quality control (QC) samples on three different concentration levels (low, medium and high) for each compound over four days. Spiked concentration was 100 (low), 1000 (medium), and 5000 (high) ng/mL for methylguanidine, *N*-acetylarginine, and indoxyl sulfate, 30 (low), 300 (medium), and 1500 (high) ng/mL for inosine and xanthurenic acid, and 50 (low), 500 (medium), and 2500 (high) ng/mL for indole-3-acetic acid. QC samples were prepared from pooled urines according to the same protocol as analyzed samples. Intra-day precision was evaluated from five repeated injections over one day while inter-day assay evaluating three replicates for four consecutive days. Precision was expressed as relative standard deviations (RSD). Recoveries for the evaluation of different sample preparation methods were calculated using pooled urine sample spiked with a standard solution at three concentration levels (low, medium, high). The calculated concentration of metabolites in urine were compared with concentrations of a standard solution in starting mobile phase. The matrix effect was calculated as a percentual decrease of standard intensity when analyzed in a sample matrix—urine over its intensity in starting mobile phase composition. Internal standards were used for this purpose. Autosampler stability was evaluated after 24h storage at 10 °C. Freeze/thaw stability was determined by comparing the recoveries of fresh QC samples and samples from the same batch frozen to −20 °C and thawed after 24 h in three cycles.

### 3.8. Data Processing and Statistical Analysis

The statistical analyses were performed in R programming environment [32]. Due to the presence of outliers, the data were winsorised using Tukey interquartile range approach [33] for each metabolite separately for ASD children and age-matched controls in pre-school and school children, resp., then for each dietary restriction and gastrointestinal problem in ASD children. These winsorised data were used in the visualization as boxplots and in statistical inference. The nullity of differences in means were assessed using a two-sample Student *t*-test with Welch approximation of degrees of freedom [34]. All alternative hypotheses were two-sided and statistical tests were performed at a significance level equal to 0.05.

## 4. Conclusions

In this study, we aimed to analyze the levels of metabolites that were proposed as potential urinary markers for ASD. For this purpose, we have developed and validated a fast and sensitive LC/MSMS method for exact quantification of these metabolites. We have applied this method for the analysis of urine samples from Slovak children with ASD and typically developing children. This analysis revealed elevated levels of markers indicating altered oxidative status and dysregulated gut microbiota. Changes were found mainly in school children. Our results provide insights into the molecular processes altered in ASD and highlights the use of urine as a source of useful disease markers. The proposed method could be used for the quantification of these six markers in human urine for further studies of the ASD metabolome.

## Figures and Tables

**Table 1:** Multi-reaction mode transition settings.

Compound Name	Ion Mode	Parent Ion (m/z)	Daughter Ion (m/z)	Cone Voltage (V)	Collision Energy (eV)
Methylguanidine	+	74.15	57.11	20	10
Methylguanidine-D3	+	76.90	60.10	25	15
*N*-acetylarginine	+	217.05	158.02	30	20
*N*-acetylarginine-D7	+	223.14	164.02	30	20
Inosine	-	267.10	135.00	40	20
Inosine-N4	-	270.93	138.91	40	25
Xanthurenic Acid	-	203.79	160.03	25	20
Xanthurenic Acid-D4	-	207.93	163.84	25	20
Indoxyl sulphate	-	212.00	132.02	30	20
Indoxyl sulphate-C6	-	217.85	137.99	30	25
Indole-3-acetic acid	-	174.04	130.16	20	10
Indole-3-acetic acid-D7	-	180.98	137.00	20	10

**Table 2:** Linearity parameters, the limit of quantification and limit of detection.

Analyte	Retention Time (min)	R^2^	Regression Equation	Linear Range ^a^	LOQ ^a^	LOD ^a^
Methylguanidine	0.63	0.99958	y = 0.000168985x − 0.000447236	40–8000	40	11.0
*N*-acetylarginine	0.71	0.99982	y = 0.000639819x + 0.000866957	160–16,000	160	12.0
Inosine	1.00	0.99980	y = 0.00170719x + 0.000559034	12–2400	12	1.1
Xanthurenic acid	2.76	0.99879	y = 0.00182916x + 0.00182916	12–2400	12	1.5
Indoxyl sulphate	2.92	0.99977	y = 0.000522458x + 0.00146806	480–48,000	480	15.8
Indole-3-acetic acid	3.87	0.99990	y = 0.00157307x + 0.00153278	20–4000	20	1.9

^a^ in ng/mL.

**Table 3:** Urine concentrations of the metabolites in the pre-school children (<6 years) and school-children (≥6 years).

**<6 Years**	**CTRL**			**ASD**			
	**Mean ^a^**	**SD**	**Median ^a^**	**Mean ^a^**	**SD**	**Median ^a^**	***p*-val**
Methylguanidine	0.50	0.14	0.48	0.52	0.21	0.46	0.759
*N*-acetyl arginine	5.31	2.44	4.81	6.02	3.45	5.24	0.395
Indole-3-acetic acid	1.34	1.11	0.93	1.23	0.77	1.04	0.741
Indoxyl sulphate	45.81	22.89	38.18	37.22	24.38	29.62	0.228
Xanthurenic acid	0.36	0.18	0.30	0.29	0.16	0.25	0.172
Inosine	0.28	0.10	0.26	0.31	0.14	0.28	0.300
**≥6 Years**	**CTRL**			**ASD**			
	**Mean ^a^**	**SD**	**Median ^a^**	**Mean ^a^**	**SD**	**Median ^a^**	***p*-val**
Methylguanidine	0.40	0.13	0.41	0.55	0.28	0.47	0.045
*N*-acetyl arginine	4.14	2.94	2.41	6.29	2.48	6.25	0.036
Indole-3-acetic acid	0.54	0.34	0.44	0.86	0.55	0.78	0.035
Indoxyl sulphate	18.95	7.11	17.49	32.63	10.38	30.15	0.00004
Xanthurenic acid	0.22	0.09	0.20	0.24	0.10	0.22	0.533
Inosine	0.24	0.11	0.20	0.32	0.16	0.31	0.062

^a^ in µmol/mmol Cr.

## Supplementary Materials

The following are available online at https://www.mdpi.com/2218-1989/10/11/443/s1, Figure S1: Representative MRM ion-chromatograms of different columns—HILIC (amide—A, diol—B), RP (CSH18—C, Cortecs T3—D, Shield RP18—E, Luna Polar C18—F), Figure S2: Comparison of intensities (A) and signal to noise ratios (B) of two C18 columns tested under reverse phase mode, Figure S3: Comparison of relative intensities of compounds in spiked urine samples after Solid phase extraction (black) and freeze-drying (grey), Table S1: Recoveries after different sample preparation procedures, Table S2: Changes of the signal intensity after 1:1 and 1:2 sample dilution, Table S3: Intra-day and inter-day accuracy and precision statistics from analysis of QC samples, Table S4: Matrix effects, Table S5: The autosampler stability, Table S6: Statistical evaluation of the influence of dietary restrictions and gastrointestinal problems on metabolite levels.
