# Supplementary material for: A Novel UHPLC-MS Method Targeting Urinary Metabolomic Markers for Autism Spectrum Disorder
Source: Metabolites. 2020 Nov 2;10(11):443. doi: 10.3390/metabo10110443 (PMC7693535; doi:10.3390/metabo10110443)
Supplement: Supplementary file 1 [file metabolites-10-00443-s001.pdf]

# A novel UHPLC-MS method targeting urinary metabolomic markers for Autism Spectrum disorder

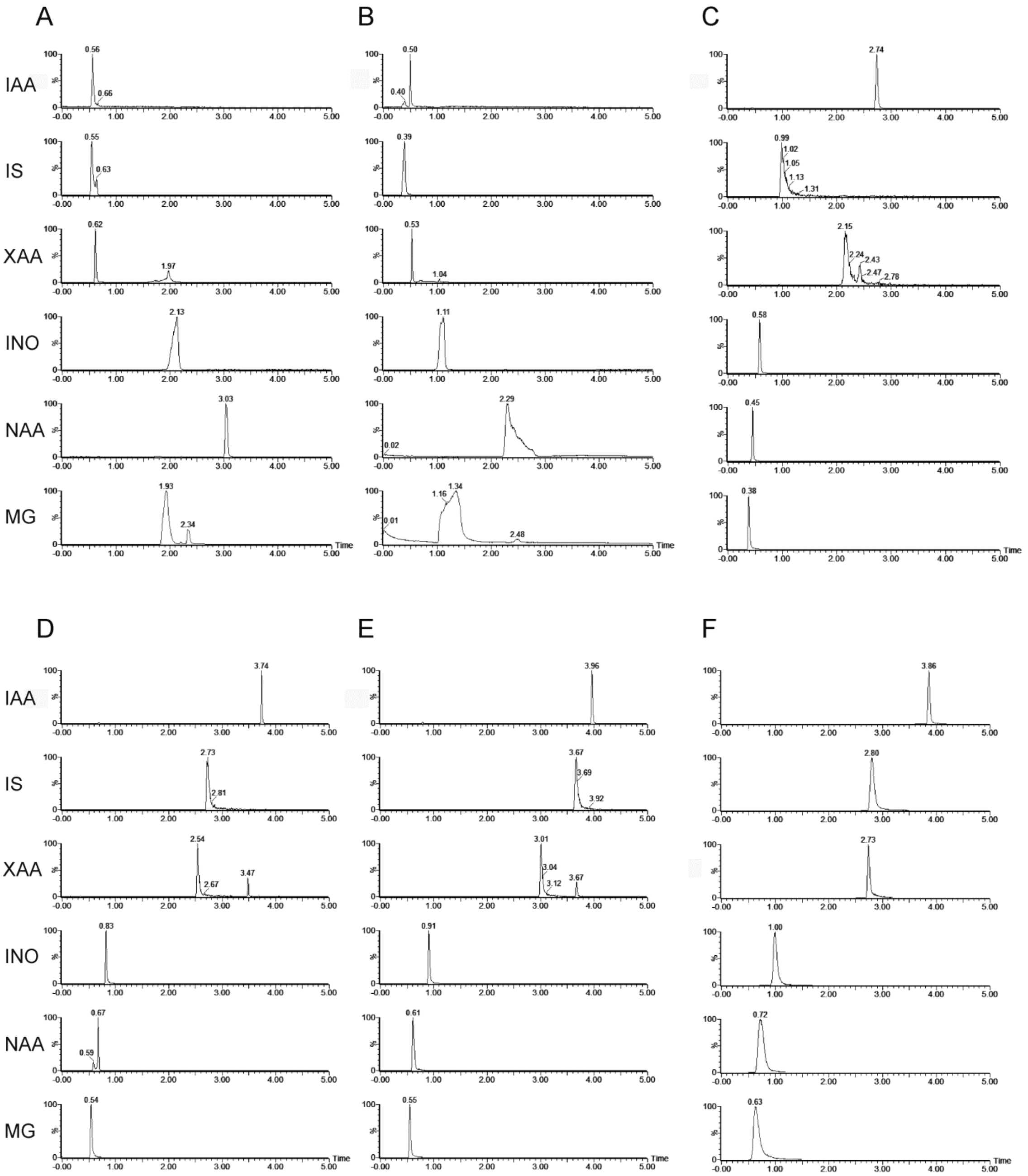


**Figure S1** - Representative MRM ion-chromatograms of different columns – **HILIC** (amide – A, diol - B), **RP** (CSH18 – C, Cortecs T3 – D, Shield RP18 – E, Luna Polar C18 - F)

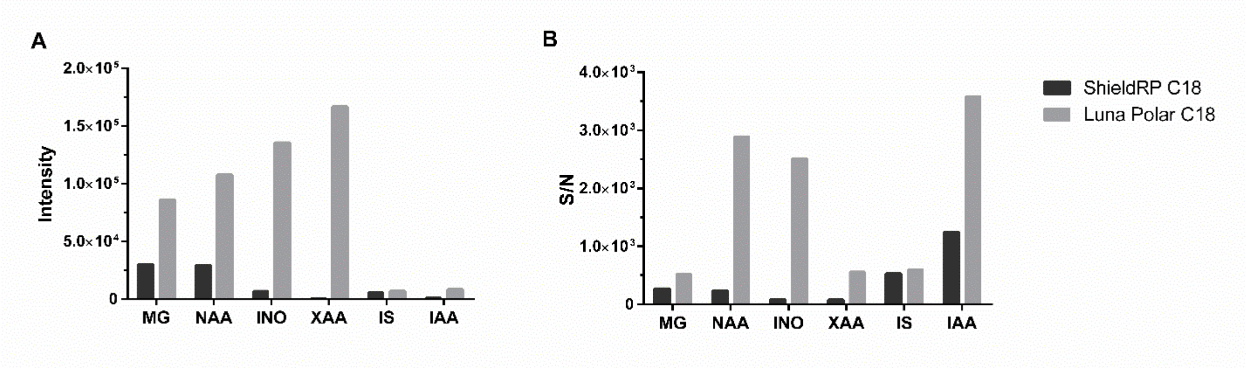


**Figure S2** - Comparison of intensities (A) and signal to noise ratios (B) of two C18 columns tested under reverse phase mode

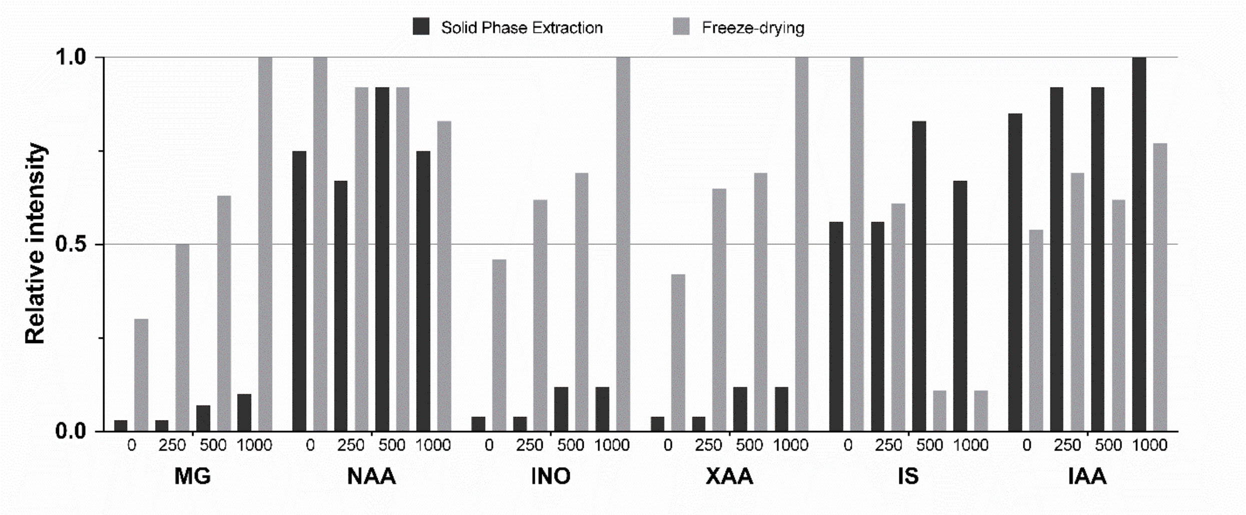


**Figure S3** - Comparison of relative intensities of compounds in spiked urine samples after Solid phase extraction (black) and freeze-drying (grey)

**Table S1** – Recovery after different sample preparation procedures

| Compound | Recovery (%) | | |
|---|---|---|---|
| | **OASIS MCX** | **OASIS HLB** | **Dilution 1:2** |
| Methylguanidine | 45.7 | 69.6 | 91.1 |
| Indole-3-acetic acid | 80.9 | 102.3 | 109.2 |
| N-acetylarginine | 43.5 | 96.3 | 104.0 |
| Indoxyl sulphate | 7.9 | 81.8 | 108.7 |
| Xanthurenic Acid | 99.5 | 110.2 | 96.5 |
| Inosine | 109.3 | 99.3 | 101.8 |

**Table S2** – Changes of the signal intensity after 1:1 and 1:2 sample dilution

| **Compound** | **Intensity** (AUC) | | **Δ%** |
|---|---|---|---|
| | **1:2** | **1:1** | |
| Methylguanidine | 2 302.5 | 2 303.1 | 0.03 |
| N-acetylarginine | 136 173.0 | 158 869.2 | 16.67 |
| Inosine | 448.3 | 428.6 | -4.38 |
| Xanthurenic Acid | 901.9 | 1 088.1 | 20.65 |
| Indoxyl sulphate | 32 885.4 | 39 852.8 | 21.19 |
| Indole-3-acetic acid | 3 986.7 | 4 526.9 | 13.55 |

**Table S1** - Intra-day and inter-day accuracy and precision statistics from analysis of QC samples.

| | | **Intra-day (n= 5)** | | | **Inter-day (n= 12)** | | |
|---|---|---|---|---|---|---|---|
| **Analyte** | **Spiked conc.**[a] | **Mean conc.**[a] | **RSD** (%) | **Accuracy** (%) | **Mean conc.**[a] | **RSD** (%) | **Accuracy** (%) |
| Methylguadinine | 5000 | 3614.0 | 0.3 | 72.3 | 3807.0 | 13.3 | 76.1 |
| | 1000 | 697.4 | 0.9 | 69.7 | 702.5 | 10.7 | 70.2 |
| | 100 | 60.8 | 1.1 | 60.8 | 61.1 | 12.4 | 61.1 |
| N-acetylarginine | 5000 | 5080.7 | 1.7 | 101.6 | 5251.3 | 2.2 | 105.0 |
| | 1000 | 1000.8 | 0.6 | 100.1 | 1004.6 | 4.3 | 100.5 |
| | 100 | 99.0 | 5.9 | 99.0 | 95.9 | 8.0 | 95.9 |
| Inosine | 1500 | 1491.2 | 2.8 | 99.4 | 1506.9 | 3.0 | 100.5 |
| | 300 | 307.2 | 2.4 | 102.4 | 304.0 | 5.4 | 101.3 |
| | 30 | 31.9 | 10.1 | 106.3 | 28.9 | 14.8 | 96.4 |
| Xanthurenic acid | 1500 | 1534.9 | 1.2 | 102.3 | 1489.0 | 11.5 | 99.3 |
| | 300 | 302.6 | 3.6 | 100.9 | 282.4 | 10.0 | 94.1 |
| | 30 | 29.6 | 3.6 | 98.6 | 27.4 | 11.6 | 91.4 |
| Indoxyl sulphate | 5000 | 4952.8 | 0.8 | 99.1 | 4700.61 | 6.1 | 94.0 |
| | 1000 | 978.3 | 2.8 | 97.8 | 979.91 | 5.1 | 98.0 |
| | 100 | 91.8 | 9.3 | 91.8 | 90.29 | 13.2 | 90.3 |
| Indole-3-acetic acid | 2500 | 2471.4 | 0.7 | 98.9 | 2557.7 | 4.5 | 102.3 |
| | 500 | 466.2 | 1.4 | 93.2 | 488.1 | 5.9 | 97.6 |
| | 50 | 46.3 | 6.3 | 92.6 | 44.2 | 11.9 | 88.5 |

[a] *concentration in ng/ml*

**Table S4** - Matrix effects

| Analyte | **Matrix effect** (%) |
|---|---|
| Methylguanidine | 74 |
| N-acetylarginine | 68 |
| Inosine | 89 |
| Xanthurenic acid | 38 |
| Indoxyl sulphate | 17 |
| Indole-3-acetic acid | 57 |

**Table S5** - The autosampler stability

| Compound | | Mean concentration[a] | Mean concentration[a] after 24h | Change % | Mean concentration[a] after three freeze/thaw cycles | Change % |
|---|---|---|---|---|---|---|
| Methylguadinine | QC 0 | 92.4 | 95.1 | 2.92 | 89.85 | -2.71 |
| | QC L | 158.7 | 158.5 | -0.13 | 161.55 | 1.83 |
| | QC M | 894.4 | 889.7 | -0.53 | 887.6 | -0.75 |
| | QC H | 4151.3 | 4128.2 | -0.56 | 4215.9 | 1.56 |
| N-acetylarginine | QC 0 | 1426.6 | 1436.2 | 0.68 | 1427.7 | 0.08 |
| | QC L | 1533.1 | 1530.2 | -0.19 | 1530.7 | -0.16 |
| | QC M | 2397.4 | 2506.4 | 4.55 | 2476.0 | 3.28 |
| | QC H | 6734.5 | 6762.8 | 0.42 | 6674.1 | -0.90 |
| Inosine | QC 0 | 272.4 | 239.6 | -12.02 | 277.9 | 2.02 |
| | QC L | 293.4 | 271.1 | -7.62 | 303.5 | 3.44 |
| | QC M | 593.7 | 578.3 | -2.60 | 605.6 | 2.00 |
| | QC H | 1779.0 | 1776.7 | -0.13 | 1784.7 | 0.32 |
| Xanthurenic acid | QC 0 | 70.5 | 72.0 | 2.20 | 84.4 | 19.80 |
| | QC L | 94.0 | 95.2 | 1.28 | 111.2 | 18.36 |
| | QC M | 361.4 | 367.5 | 1.69 | 383.0 | 5.98 |
| | QC H | 1710.3 | 1743.8 | 1.96 | 1872.7 | 9.50 |
| Indoxyl sulphate | QC 0 | 4082.5 | 4076.3 | -0.15 | 4903.1 | 20.10 |
| | QC L | 4176.3 | 4145.2 | -0.74 | 4989.2 | 19.47 |
| | QC M | 5054.8 | 5126.0 | 1.41 | 5918.2 | 17.08 |
| | QC H | 8301.6 | 8271.3 | -0.36 | 9852.7 | 18.68 |
| Indole-3-acetic acid | QC 0 | 185.2 | 171.7 | -7.29 | 172.7 | -6.78 |
| | QC L | 221.1 | 216.7 | -1.99 | 211.0 | -4.57 |
| | QC M | 700.5 | 691.8 | -1.23 | 700.3 | -0.02 |
| | QC H | 2833.6 | 2892.7 | 2.09 | 2905.8 | 2.55 |

[a] *concentration in ng/ml*

**Table S6** - Statistical evaluation of the influence of dietary restrictions (0-no restrictions, 1-restrictions) and gastrointestinal problems (0-no GIT problems, 1-GIT problems) on metabolite levels.

| **Dietary restrictions** | | **0** | | | **1** | | |
|---|---|---|---|---|---|---|---|
| | Mean [a] | SD | Median [a] | Mean [a] | SD | Median [a] | p-value |
| Methylguanidine | 0.55 | 0.27 | 0.45 | 0.51 | 0.21 | 0.54 | 0.615 |
| N-acetyl arginine | 5.96 | 2.43 | 5.73 | 7.57 | 3.66 | 6.85 | 0.181 |
| Indole-3-acetic acid | 0.99 | 0.50 | 0.86 | 1.35 | 1.03 | 1.18 | 0.260 |
| Indoxyl sulphate | 35.88 | 16.33 | 30.73 | 41.13 | 19.30 | 32.19 | 0.417 |
| Xanthurenic acid | 0.26 | 0.14 | 0.24 | 0.29 | 0.12 | 0.23 | 0.528 |
| Inosine | 0.32 | 0.15 | 0.27 | 0.29 | 0.11 | 0.29 | 0.490 |

[a] *in μmol/mmol Cr*

| **GI problems** | | **0** | | | **1** | | |
|---|---|---|---|---|---|---|---|
| | Mean [a] | SD | Median [a] | Mean [a] | SD | Median [a] | p-val |
| Methylguanidine | 0.47 | 0.21 | 0.42 | 0.57 | 0.27 | 0.58 | 0.183 |
| N-acetyl arginine | 6.46 | 3.40 | 6.06 | 6.44 | 2.56 | 6.08 | 0.984 |
| Indole-3-acetic acid | 1.16 | 0.88 | 0.76 | 1.01 | 0.47 | 0.97 | 0.532 |
| Indoxyl sulphate | 32.47 | 14.60 | 27.38 | 40.99 | 18.51 | 35.57 | 0.105 |
| Xanthurenic acid | 0.29 | 0.13 | 0.26 | 0.25 | 0.13 | 0.23 | 0.381 |
| Inosine | 0.28 | 0.13 | 0.23 | 0.34 | 0.14 | 0.31 | 0.156 |

[a] *in μmol/mmol Cr*
